# Identification of Candidate Gene Regions in the Rat by Co-Localization of QTLs for Bone Density, Size, Structure and Strength

**DOI:** 10.1371/journal.pone.0022462

**Published:** 2011-07-27

**Authors:** Sofia Lagerholm, Hee-Bok Park, Holger Luthman, Marc Grynpas, Fiona McGuigan, Maria Swanberg, Kristina Åkesson

**Affiliations:** 1 Clinical and Molecular Osteoporosis Unit, Department of Clinical Sciences Malmö, Lund University, Lund, Sweden; 2 Medical Genetics Unit, Department of Clinical Sciences Malmö, Lund University, Lund, Sweden; 3 Department of Orthopedics, Skåne University Hospital Malmö, Malmö, Sweden; 4 Institute of Biomaterials and Biomedical Engineering, University of Toronto and Samuel Lunenfeld Research Institute of Mount Sinai Hospital, Toronto, Canada; The University of Hong Kong, Hong Kong

## Abstract

Susceptibility to osteoporotic fracture is influenced by genetic factors that can be dissected by whole-genome linkage analysis in experimental animal crosses. The aim of this study was to characterize quantitative trait loci (QTLs) for biomechanical and two-dimensional dual-energy X-ray absorptiometry (DXA) phenotypes in reciprocal F2 crosses between diabetic GK and normo-glycemic F344 rat strains and to identify possible co-localization with previously reported QTLs for bone size and structure. The biomechanical measurements of rat tibia included ultimate force, stiffness and work to failure while DXA was used to characterize tibial area, bone mineral content (BMC) and areal bone mineral density (aBMD). F2 progeny (108 males, 98 females) were genotyped with 192 genome-wide markers followed by sex- and reciprocal cross-separated whole-genome QTL analyses. Significant QTLs were identified on chromosome 8 (tibial area; logarithm of odds (LOD) = 4.7 and BMC; LOD = 4.1) in males and on chromosome 1 (stiffness; LOD = 5.5) in females. No QTLs showed significant sex-specific interactions. In contrast, significant cross-specific interactions were identified on chromosome 2 (aBMD; LOD = 4.7) and chromosome 6 (BMC; LOD = 4.8) for males carrying F344mtDNA, and on chromosome 15 (ultimate force; LOD = 3.9) for males carrying GKmtDNA, confirming the effect of reciprocal cross on osteoporosis-related phenotypes. By combining identified QTLs for biomechanical-, size- and qualitative phenotypes (pQCT and 3D CT) from the same population, overlapping regions were detected on chromosomes 1, 3, 4, 6, 8 and 10. These are strong candidate regions in the search for genetic risk factors for osteoporosis.

## Introduction

Osteoporosis is a common multifactorial disorder characterized by reduced bone mineral density (BMD) and compromised bone quality, with micro-architectural deterioration leading to an increased susceptibility to fracture [1]. The ability of bone to resist fracture is determined by several highly heritable phenotypes including BMD, bone size, bone structure and strength [2], [3], [4]. Identification of genes and pathways regulating these phenotypes and thereby underlying bone strength could provide valuable insight regarding susceptibility to osteoporosis and fracture risk.

Translational genetics starting with experimental models is a valuable tool to dissect complex genetic traits. By using inbred strains and crosses between strains, genetic heterogeneity is dramatically reduced and experimental tests, such as destructive testing of bone, can be performed. One example of successful translational genetics is the identification of the arachidonate lipoxygenase 15 (Alox15) gene encoding an enzyme that modifies polyunsaturated fatty acids, as a candidate for osteoporosis. Mice deficient in Alox15 were found to have increased bone mass [5] and subsequent association analyses in humans have shown association with BMD for SNPs in the human orthologues ALOX12 and ALOX15 [6], [7].

Most experimental genetic studies on bone have been performed in the mouse [8], [9], [10], [11], [12], but observed variations in BMD, bone structure, and fragility between different inbred rat strains [13], together with progress in mapping the rat genome has established the rat as a useful genetic model for skeletal fragility [14], [15], [16]. Rat models offer several advantages in studying biomechanical phenotypes reflecting the ability of bone to resist fracture, since the larger bone size of rats compared to mice allows greater precision of both structural and biomechanical measurements. The biomechanical three-point bending test (3PB) used in this study provides clinically relevant phenotypes comparable to fracture in humans [17]. Fracture susceptibility is affected by a number of conditions including diabetes, where hyperinsulinemia has anabolic effects and hypoinsulinemia is associated with bone loss and increased risk of fracture [18], [19], [20], [21], [22]. Diabetes is, however, less extensively studied with regard to fracture compared to many other conditions. The inclusion of a rat strain with diabetes-associated phenotypes in linkage analysis of bone phenotypes thus offers the possibility to identify shared mechanisms e.g. by identifying overlapping quantitative trait loci (QTLs) for these traits.

In our previous studies using reciprocal F2 crosses from inbred diabetic GK and non-diabetic F344 rats, we reported results from a genome-wide screen for size, trabecular and cortical bone structure of tibia [23], [24]. We identified several significant QTLs for both bone size and structure and additionally found evidence of sex- and reciprocal cross-specific interactions with bone traits. The reciprocal crosses differ with regard to grand-maternal origin, either GK or F344. This has epigenetic effects on the F2 offspring, but also results in the inheritance of different mitochondria. Mitochondria have small, circular genomes encoding 13 proteins taking part in the electron transport chain. The mitochondrial encoded proteins need to interact with proteins encoded by genes in the cell nucleus to function correctly. Polymorphisms in nuclear or mitochondrial DNA affecting such interactions could give rise to an observed reciprocal effect i.e. differing phenotype, depending on the combination of nuclear and mitochondrial alleles in the F2 population. Of note, the GK and F344 rat strains used differ in more than 100 positions in their mtDNA [25], [26]. The aims of this study were to identify QTLs linked to two-dimensional dual-energy X-ray absorptiometry (DXA) and bone biomechanical phenotypes in reciprocal rat F2 crosses and to combine these with previously reported QTLs for bone size and structure. This study thus addresses all the primary skeletal determinants of fracture risk including areal BMD (aBMD), bone size, structure and strength and further delineates the involvement of sex- and reciprocal cross on the genetic regulation of bone strength related phenotypes.

## Materials and Methods

### Animals

Rat strains GK/KyoSwe (GK) and F344/DuCrlSwe (F344) were maintained through brother-sister breeding. Briefly, two separate F2 intercrosses were generated: one originating from grand-maternal GK and grand-paternal F344 (cross 1, (GK female×F344 male) F1), and the other from grand-maternal F344 and grand-paternal GK (cross 2, (F344 female×GK male) F1). The two groups of reciprocal F1 progeny were mated separately to yield two reciprocal F2 populations. The rats were maintained under controlled conditions as reported previously [23]. F2 progeny were sacrificed at a mean age of 215 days and body weight and length from nose tip to tail-base were recorded. The left tibia was dissected free of fat and muscle and stored in 70% ethanol at −20°C prior to biomechanical testing and analysis of skeletal phenotypes. Genomic DNA was purified from rat liver as previously described [23]. All experiments were performed with approval from the local Animal Ethics Committee; Stockholm's norra djurförsöksetiska nämnd (N189/97), Malmö/Lunds djurförsöksetiska nämnd (M215-01 and M143-04).

### Bone strength-related phenotypes

Biomechanical testing (3PB test): Prior to testing, tibia were thawed and equilibrated at room temperature (∼2 hours). Tibia were positioned on the lower supports of a three-point bending fixture (a span of ∼16 mm) and held in a stable position by a 2N preload. Using an Instron 4465 materials testing machine (Instron Inc., Canton, MA, USA) with a 1KN load cell, the bones were loaded at their midpoint at a deformation rate of 1 mm/min until fracture. Load-displacement data representing structural or extrinsic properties of the bone were calculated from load-displacement curves and collected using LABView data acquisition software [27]. Parameters included: ultimate force (N; height of curve) reflecting the maximum load the bone can absorb before failing (i.e. bone strength), stiffness (N/mm; initial slope of the load displacement curve), and work to failure (mJ; area under curve) reflecting the total energy the bone can absorb before fracture.

Prior to mechanical testing, whole tibiae were scanned by DXA using the PIXImus™ densitometer (GE Lunar, Madison, WI). Bones were precisely positioned at the center of the X-ray cone beam and scanned in air on the Plexiglas platform provided. A grid on the Plexiglas platform allowed the position of each tibia to remain consistent between samples. aBMD was automatically calculated from the bone mineral content (BMC) and the user-defined measured region of bone area [28]. The instrument was calibrated daily using the manufacturer's phantom. Additionally, we refer to previously reported phenotypes obtained from three-dimensional computed tomography (3D CT) and peripheral quantitative CT (pQCT) [23], [24].

### Statistical analysis

All phenotypes were normally distributed or log-transformed to obtain a normal distribution. To compare the bone phenotypes between males and females and between the reciprocal crosses, one-way ANOVA was used. The level of significance was set at *p*<0.05. Unless stated, *p*-values are nominal. Phenotypes were adjusted for reciprocal cross, age, litter size, and body-weight using regression analyses. Residuals were checked for normality and used in the QTL analysis. To account for gender attributed bone quality differences, the residuals were computed separately for each sex. Correlations between all measured bone phenotypes in the F2 progeny were evaluated using Pearson's correlation coefficients.

### Genotyping and linkage analysis of quantitative traits

A total of 192 genome-wide microsatellite markers at a spacing of 10 cM, were genotyped in the F2 progeny (108 males, 98 females) and a genetic map was generated as described previously [23]. Linkage analysis was performed for each sex separately. In order to identify possible interaction differences between loci in the nuclear genome and mitochondrial DNA, the sex separated F2 progeny were also separated on the basis of reciprocal cross. QTLs on autosomes were identified employing MAP MANAGER/QTX v. b20 [29]. R/qtl was used to include the X chromosome in the linkage analysis and to confirm all reported autosomal QTLs [30]. Mapping QTLs on the X chromosome was conducted for each sex separately without further separation by cross. Permutation tests were performed to establish genome-wide significance levels by randomization of the phenotypic data in relation to genotypic data [31]. Significant (i.e. genome-wide false-positive rate of <5%) and suggestive (i.e. genome-wide false-positive rate of <63%) linkage was employed to establish genome-wide thresholds [32], [33]. The likelihood ratio (LR) for suggestive linkage was 10.7 (logarithm of odds (LOD) = 2.3), and for significant linkage the LR range was 17.4–17.7 (LOD = 3.8). The approximate size of the QTL was defined as the region covered by a 1-LOD reduction for any of the bone traits.

### Evaluation of sex- and reciprocal cross specific QTLs

Evaluation of sex- and reciprocal cross specific QTLs were performed for QTLs that reached significant linkage (LOD≥3.8), following the method described previously [23]. To identify sex-specific QTLs, the LOD score differences between males and females across the genome were assessed (ΔLOD_sex_ score). We applied a permutation method to evaluate sex-specific QTLs, where thresholds were established using two randomly selected equal sized subsets of males and females [34]. The randomization was conducted within each cross. Subsequently, the bone phenotypes in the two subsets were permutated to calculate ΔLOD_sex_ scores across the genome. Genetic markers on the X chromosome were not included in the permutation tests. The average ΔLOD_sex_ score for genome-wide significant sex-specificity at suggestive (α = 0.63) and significant (α = 0.05) level were 2.3 and 3.7, respectively.

Within each sex, subsequent reciprocal cross-separated linkage analyses were conducted to identify cross-specific QTLs. The LOD score differences between cross 1 and cross 2 (ΔLOD_cross_ score) across the genome were evaluated. Thresholds of the cross specific QTLs were computed by permutation using two randomly selected equal sized cross 1 and cross 2 subsets. The average ΔLOD_cross_ score for genome-wide significant cross-specificity at suggestive (α = 0.63) and significant (α = 0.05) level were 2.4 and 3.9, respectively.

To confirm the sex- and cross-specific QTLs identified with the ΔLOD method, likelihood ratio tests were performed comparing a full model containing a QTL×sex interaction term/cross interaction term and a reduced model without the interaction term. Both models used male and female data for sex interaction and data from the two crosses in each sex for cross interaction.

Residuals of each phenotype were examined for normality using normal probability plots. The level of significance for a specific QTL interaction with sex or cross was set at *p*<0.05.

A statistical power calculation for sample size, using the method of Lynch and Walsh [35] was performed as described previously [23]. Using a LOD score of 2.4 to control false positive detection of linkage, a sample size of 52 is necessary to achieve 80% statistical power for detecting a QTL with R2 value (i.e. fraction of phenotypic variance explained) of 0.25.

## Results

### Influence of sex and reciprocal cross on biomechanics and DXA

Strong sexual dimorphism was observed for all biomechanical and DXA traits in the F2 progeny with significantly higher mean values in males. In contrast, no differences were observed between phenotypic mean values in the two reciprocal F2 crosses (Table 1).

**Table 1:** Biomechanical and DXA data from tibia of female and male F2 progeny generated from GK and F344 in two reciprocal crosses.

	Sex Effects			Reciprocal Cross Effects					
	Irrespective of cross			Females			Males		
	Females (n = 98)	Males (n = 108)	% Difference (p)	Cross 1 (n = 48)	Cross 2 (n = 50)	% Difference (p)	Cross 1 (n = 66)	Cross 2 (n = 42)	% Difference (p)
**General Characteristics**									
Body weight (g)	248±27	426±44	−42 (<10^−4^)	247±25	248±28	−0.4 (NS)	423±44	423±45	−0.05 (NS)
Body length (cm)	21.3±0.7	24.9±0.8	−14 (NS)	21.3±0.7	21.2±0.8	0.5 (NS)	24.9±0.8	24.9±0.7	∼0 (NS)
Tibia length (mm)	38.9±1.3	43.8±1.3	−11 (<10^−4^)	39.1±1.2	38.7±1.3	1.0 (NS)	43.9±1.3	43.6±1.2	0.7 (NS)
**3- Point Bending Test**									
Ultimate force (N)	73.4±10.0	118±15.3	−38(<10^−4^)	73.4±9.4	73.3±10.6	0.1 (NS)	118±15.3	118±15.5	∼0 (NS)
Work to failure (mJ)	42.8±12.0	73.6±27.3	−42(<10^−4^)	44.3±13.6	41.4±10.3	7.0 (NS)	72.6±26.7	75.1±28.4	−3.3 (NS)
Stiffness (N/mm)	205±35.6	340±65.8	−40(<10^−4^)	207±35.4	204±36.0	1.5 (NS)	334±56.6	348±78.3	−4.0 (NS)
**DXA**									
aBMD (g/cm^2^)	0.16±0.01	0.18±0.01	−11 (<10^−4^)	0.16±0.01	0.16±0.01	∼0 (NS)	0.18±0.008	0.18±0.009	∼0 (NS)
BMC (g)	0.31±0.04	0.44±0.05	−30(<10^−4^)	0.31±0.03	0.30±0.04	3.3 (NS)	0.44±0.05	0.44±0.05	∼0 (NS)
Projected area (cm^2^)	1.92±0.14	2.46±0.17	−22(<10^−4^)	1.94±0.13	1.90±0.15	2.1 (NS)	2.46±0.18	2.47±0.16	−0.4 (NS)

Phenotypes are uncorrected and presented as mean ± sd. Cross 1 originate from grand-maternal GK- and cross 2 from grand-maternal F344 rats.

Percentage difference (female compared to male or cross 1 compared to cross 2) is indicated, and nominal *p*-values determined by ANOVA are given when *p*<0.05.

### QTLs linked to biomechanics

**Figure 1:**
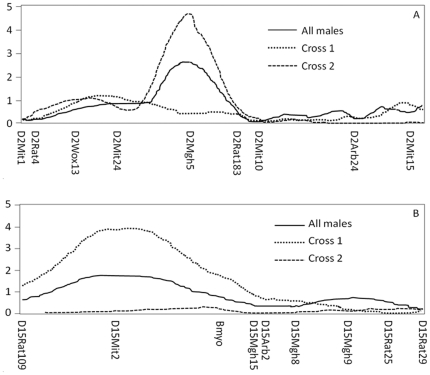
Cross-specific QTLs in males. (A) QTL for aBMD on chromosome 2 and (B) QTL for ultimate force on chromosome 15. Cross 1 represents males with GKmtDNA and cross 2 represents males with F344mtDNA.

Results from the sex- and reciprocal cross separated QTL analyses for biomechanical phenotypes of tibia are summarized in Table 2. No significant linkage to biomechanical properties was identified in males when both reciprocal crosses were combined. When separating males by reciprocal cross, a QTL on chromosome 15 linked to ultimate force reached genome-wide significance in cross 1 with GK grand-maternal origin. This QTL met the criteria for genome-wide cross-specificity at the suggestive level (ΔLOD_cross_≥2.4), and the likelihood ratio test confirmed a cross-specific interaction for this locus (LR = 10.4, *p* = 0.005) (Fig. 1B). An overlapping QTL in females from cross 2 was suggestively linked to work to failure (Table 2).

**Table 2:** QTLs linked to biomechanics and DXA phenotypes in male and female F2 rats. Suggestive QTLs are reported if overlapping a significant QTL.

					LOD scores		
	Chr	QTL region	Position (cM)	Phenotype	Male (n = 108)	Cross 1 (n = 66)	Cross 2 (n = 42)
**Biomechanics**	15	D15Rat109-D15Mit2	10–32	Ult force	1.8	3.9^b^^,^ c	0.2
**DXA**	2	D2Mit24-D2Mgh5	45–59	BMC	2.2	0.4	2.8^a^
	2	D2Mit24-D2Mgh5	45–57	aBMD	2.5^a^	0.8	4.7^b^^,^ d
	6	D6Mgh11-D3Mit19	25–58	BMC	2.5^a^	0.8	4.8^b^^,^ d
	6	D6Mgh11-D3Mit19	30–60	Area	2.8^a^	1.3	3.1^a^
	8	D8Mit3-D8Mit2	35–58	BMC	4.1^b^	1.3	2.9^a^
	8	D8Mit2-D8Mgh4	43–59	Area	4.7^b^	1.4	3.3^a^

					Female (n = 98)	Cross 1 (n = 48)	Cross 2 (n = 50)
**Biomechanics**	1	D1Rat7-D1Mit9	17–40	Ult force	3.6^a^	2.5^a^	2.5^a^
	1	D1Rat7-D1Mit9	18–43	Stiffness	5.5^b^	4.7^b^	3.1^a^
	15	D15Mit2-Bmyo	25–37	Work to fail	0.5	0.1	2.8^a^
**DXA**	4	D4Mit9-D4Mit24	37–49	aBMD	1.8	3.7^a^	0.3
	4	D4Mit9-D4Mit24	36–48	BMC	1.7	5.1^b^	0.3
	X	DXRat20-DXRat103	52–74	Area	3.9^b^		

QTL size defined as the region covered by a 1-LOD reduction for any of the bone traits.

a Genome-wide suggestive QTL (LOD≥2.3).

b Genome-wide significant QTL (LOD≥3.8).

c Suggestive cross-specific QTLs (ΔLODcross≥2.4) validated by likelihood ratio (LR) tests for QTL-by-cross interaction (*p*<0.05).

d Significant cross-specific QTLs (ΔLODcross≥3.9) validated by likelihood ratio (LR) tests for QTL-by-cross interaction (p<0.05).

The maximum LOD-score for biomechanical phenotypes in females was 5.5 on chromosome 1 (18–43 cM) with significant linkage to stiffness. This locus also showed linkage to ultimate force at the suggestive level. The chromosome 1 QTL linked to stiffness reached genome-wide significance for female-specific interaction with ΔLOD_sex_ evaluation (ΔLOD_sex_≥3.7, results not shown) but did not reach significance in the likelihood ratio test. Suggestive QTLs for biomechanical phenotypes are reported in table S1.

### QTLs linked to DXA phenotypes

Results from the sex- and reciprocal cross separated QTL analysis for DXA traits are summarized in Table 2. In all males, significant linkage to BMC and to area was identified on chromosome 8 (35–59 cM). The chromosome 8 BMC QTL indicated male-specific interaction (ΔLOD_sex_>2.3, results not shown) but was not confirmed in the likelihood ratio test. In males with F344 grand maternal origin (cross 2), significant linkage was detected on chromosome 2 for aBMD, and on chromosome 6 for BMC. The QTL on chromosome 2 also overlapped with a suggestive QTL linked to BMC in cross 2 males. The significant QTLs on chromosome 2 (aBMD) (Fig. 1A) and 6 (BMC) reached genome-wide significance in the ΔLOD_cross_ evaluation (ΔLOD_cross_≥3.9) and cross-specific interactions were confirmed for both by likelihood ratio tests (LR = 10.4, *p* = 0.005 and LR = 10.9, *p* = 0.004) respectively.

In females, QTL analysis on the X chromosome without separation by cross identified a significant QTL linked to tibial area. The cross-separated QTL analysis revealed significant linkage to BMC on chromosome 4 (36–48 cM) in females with GK grand maternal origin (cross 1). An overlapping region also showed suggestive linkage to aBMD in cross 1. The QTL identified on chromosome 4 significantly linked to BMC reached genome-wide significance in the ΔLOD_cross_ evaluation (ΔLOD_cross_≥3.9), but did not reach significance for cross-specific interaction in the likelihood ratio test. Suggestive QTLs for DXA phenotypes are reported in table S1.

### Co-allelic effects

The GK allele had an increasing effect on genotypic mean values for the majority of the QTLs significantly linked to both biomechanical and DXA traits in males and females (Table 3). There were however also heterosis-like effects, where the heterozygous group carrying one GK- and one F344 allele differed significantly from both homozygous groups and had higher mean aBMD (marker D2Mgh5) and ultimate force (marker D15Mit2).

**Table 3:** Genotypic mean values for biomechanical- and DXA phenotypes with significant linkage to chromosomes 1, 2, 4, 6, 8, 15, and X.

Chr	Peak Marker	Phenotype	GK/GK	GK/F344	F344/F344	(p-value) ANOVA
1	D1Mgh2	Stiffness^F^	243.5±31.5	199.6±34.1	184.2±25.8	7.2 E-07
1	D1Mit9	Stiffness^F,Cr1^	249.7±36.1	199.6±30.2	190.3±30.9	0.00076
2	D2Mgh5	aBMD^M,Cr2^	0.17±0.01	0.18±0.008	0.17±0.007	0.024
4	D4Mit9	BMC^F,Cr1^	0.30±0.04	0.32±0.04	0.31±0.04	NS
6	D3Mit19	BMC^M,Cr2^	0.46±0.05	0.44±0.04	0.40±0.05	NS
8	D8Mit2	BMC^M^	0.44±0.04	0.45±0.04	0.43±0.05	NS
8	D8Mit2	Area^M^	2.45±0.13	2.50±0.16	2.40±0.19	NS
15	D15Mit2	Ult force^M,Cr1^	110.8±7.94	126.8±14.3	116.3±15.6	0.0026
X	DXRat20	Area^F^	1.89±0.10	1.90±0.13	1.94±0.19	NS

Values are means ± SD. M = males; F = females; Cr1 = Cross 1; Cr2 = Cross 2.

### Co-localization of QTLs for bone strength-related phenotypes

By combining the QTLs identified in this study with QTLs linked to other bone-related phenotypes, overlapping or co-localized QTLs for biomechanical properties (3PB), bone density (DXA) and/or bone quality (pQCT and 3D CT) phenotypes were detected on chromosomes 1, 3, 4, 6, 8 and 10 (Table 4). In order to increase understanding of the observed genetic co-localizations, correlation coefficients between phenotypes mapping to the same region were evaluated. A large region on chromosome 1 (17–79 cM) displayed linkage to multiple phenotypes obtained from all four methods (3PB, DXA, pQCT and 3D CT) in both males and females. The biomechanical phenotypes ultimate force and stiffness were strongly correlated (0.64–0.88) with the other phenotypes linked to this region except for cortical volumetric BMD (cortvBMD) and metaphyseal volumetric BMD (metavBMD;−0.02–0.24). Similar patterns were seen at other loci, where most co-mapped phenotypes were correlated with each other, with exceptions mainly attributed to biomechanical phenotypes. For phenotypes linked to chromosome 3 (3–24 cM), ultimate force strongly correlated with 3D CT and pQCT phenotypes (0.71–0.88), while correlations with total bone mineral content (totBMC) and metaphyseal volumetric bone mineral density (metavBMD) were low (0.04–0.14). The QTLs that co-localized to chromosome 4 (36–74 cM) were measured by 3PB, 2D DXA and 3D CT and these phenotypes were strongly correlated (0.63–0.96) with the exception of work to failure which demonstrated low correlation with the other phenotypes (0.07–0.26). The same pattern was observed for chromosome 8 (35–63 cM) with overlapping QTLs from all four methods, but moderate correlations between work to failure and the other co-mapped phenotypes. In contrast, strong correlations (0.79–0.91) were observed for all phenotypes that co-localized to chromosome 6 (25–60 cM) and to chromosome 10 (73–91 cM) (0.45–0.93), regions where biomechanical phenotypes did not co-map.

**Table 4:** Co-localization of QTLs for biomechanics, BMD, bone size, BMC, cortical and trabecular bone traits of tibia in GKxF344 F2 rats.

	3PB (Biomechanics)			2D DXA (BMD)			3D CT (Bone size and BMC)			pQCT (Trabecular & Cortical bone)		
Chr	Phenotype	LOD score		Phenotype	LOD score		Phenotype	LOD score		Phenotype	LOD score	
Position		M/Cr1/Cr2	F/Cr1/Cr2		M/Cr1/Cr2	F/Cr1/Cr2		M/Cr1/Cr2	F/Cr1/Cr2		M/Cr1/Cr2	F/Cr1/Cr2
**1**	Ult force	2.3/**2.8**/0.9	**3.6**/2.5/2.5	aBMD		1.8/0.4/**2.7**	PAmid	3.8/**4.1**/1.7		Tibia length	**3.8**/3.0/1.7	**3.4**/2.8/0.7
17–79 cM	Stiffness	2.5/**3.5**/0.9	**5.5**/4.7/3.1	Area		**2.5**/1.3/2.3	EAfib	6.7/**7.2**/2.5	**3.8**/2.6/1.0	CortvBMD	**4.4**/3.5/0.9	
				BMC		3.2/1.5/**3.7**				MetavBMD		2.5/0.6/**4.5** c
										CortBMC	**3.6**/2.7/2.2	**3.9**/2.5/3.1
										CortCSA	**3.5**/2.9/1.8	**3.1**/2.3/2.0
										CortPC	3.2/**3.4**/0.8	**2.9**/2.0/1.9
										CortEC	2.4/**2.5**/0.3	
										IP	**3.7**/3.3/1.7	**3.5**/**3.5**/0.9
										RP	**3.7**/3.2/1.7	**3.5**/3.3/0.7
**3**	Ult force	**2.9**/2.5/1.4					PAfib		4.4/**4.8** c/1.7	CortBMC		2.2/**3.3**/0.9
3–24 cM							TotBMC		**4.3**/1.5/3.2	CortCSA		2.6/**3.6**/0.2
										CortPC		1.5/**3.1**/0.5
										RP		2.0/**3.3**/0.4
										MetavBMD		**3.6** f/1.9/2.1
**4**	Ult force		0.5/**2.5**/0.2	aBMD		1.8/**3.7**/0.3	CortBV		1.1/**4.9** c/0.1			
36–74 cM	Work to fail		0.8/**2.5**/0.3	BMC		1.7/**5.1**/0.3	TotBV		1.9/**5.6** c/0.6			
**6**				BMC	2.5/0.8/**4.8** c		TotBV	3.4/0.9/**3.8**				
25–60 cM				Area	2.8/1.3/**3.1**							
**8**	Work to fail		**2.9**/0.9/2.0	BMC	**4.1**/1.3/2.9		CortBV	3.1/0.7/**3.8**		CortPC		3.0/**3.2**/1.3
35–63 cM				Area	**4.7**/1.4/3.3					CortEC		3.1/**3.4**/1.8
										RP		**3.5**/2.8/1.6
**10**				aBMD	**2.4**/1.5/1.1		PAmid	**4.0**/2.5/1.5		CortPC		1.8/0.2/**3.1** c
73–91 cM										CortEC		2.0/0.4/**3.3** c

M = QTL detected in males; F = QTL detected in females; Cr1 = Cross 1 (GK mtDNA); Cr2 = Cross 2 (F344 mtDNA). QTLs linked to 3D-CT and pQCT phenotypes are reported in references [23], [24].

f Female-specific QTL;

c Cross-specific QTL. The strongest linkage detected in either sex or reciprocal cross is marked in bold.

CAmid = Cortical area at midshaft (mm^2^); CortBMC = cortical mineral content (mg/mm); CortBV = Cortical bone volume (mm^3^); CortvBMD = cortical volumetric BMD (g/cm^3^); CSA = Cross-sectional area (mm^2^); EAfib = Endosteal area at fibula-site (mm^2^); EC = Endosteal circumference (mm); IP = Moment of inertia (mm^4^); MetavBMD = metaphyseal volumetric BMD (g/cm^3^); PAfib = Periosteal area at fibula-site (mm^2^); PAmid = Periosteal area at midshaft (mm^2^); PC = Periosteal circumference (mm); RP = Moment of resistance (mm^3^); TotBMC = total bone mineral content (mg/mm); TotBV = Total bone volume (mm^3^).

## Discussion

In this study, we identified multiple QTLs for biomechanical and two-dimensional DXA phenotypes in F2 progeny of GK and F344 rats and confirmed bone QTL interactions with reciprocal cross. Previously, we reported results from a genome-wide screen for bone size and trabecular and cortical bone structure of tibia in the same F2 progeny [23], [24]. The biomechanical testing reported here provides bone phenotypes directly reflecting the ability of bone to resist fracture. We have thus addressed the majority of clinically important bone properties related to bone strength and fracture susceptibility.

By combining QTLs linked to distinct bone phenotypes measured by separate methods, overlapping chromosomal regions linked to bone size, structure and strength were identified. Two large regions on chromosomes 1 (17–79 cM) and 8 (35–63 cM) displayed linkage to multiple phenotypes obtained from four different methods (3PB, DXA, pQCT and 3D CT) in both males and females. The regions with overlapping QTLs influenced different phenotypes, the majority of which were correlated at a population level. Exceptions mostly represented biomechanical phenotypes. As an example, overlapping QTLs on chromosome 3 included the biomechanical phenotype ultimate force which was strongly correlated with the dimensional phenotypes 3D CT- and pQCT but only weakly correlated with totBMC and metavBMD. The co-mapping of these phenotypes was thus not due to phenotype correlation, but represents different aspects of the QTLs. Co-localization of QTLs for uncorrelated phenotypes could reflect the existence of distinct sub-loci. Conversely, highly correlated co-mapped bone phenotypes could reflect pleiotropic gene effects with dependence between phenotypes. Thus, the observed co-localizations of QTLs are unlikely to be explained by phenotypic correlation alone and the regions could each contain either a single QTL with pleiotropic effects, or several QTLs that influence the correlated phenotypes independently. Combining QTLs from different methods allows greater characterization of the gene regions, indicating whether they are likely to contain one or several candidate genes and if these are likely to participate in the same biological processes. Since the biomechanical properties of bone are the sum of many different characteristics, the lower correlation of these traits to specific quantitative and qualitative traits was expected.

The notion that QTLs for bone phenotypes interact specifically with sex have been reported previously [8] but the observed interactions between nuclear QTLs for several bone phenotypes and reciprocal crosses, that differ with regard to mitochondrial DNA sequence, demonstrates a new and important aspect to be considered when interpreting the genetics of phenotypes related to bone strength.

In this study, the reciprocal cross-separated QTL analysis in males allowed identification of cross-specific interactions for three QTLs on chromosomes 2 (aBMD), 6 (BMC) and 15 (ultimate force). The QTLs on chromosomes 2 and 6 were identified in all males at a suggestive level but showed genome-wide significant linkage only in the cross with F344 grand maternal origin, while the QTL on chromosome 15 was only detected in the cross with GK grand maternal origin and would not have been detected in the combined sample including both reciprocal crosses. This illustrates the importance of sub-group analysis and of considering reciprocal cross effects in order to improve the detection of candidate genes for fracture susceptibility.

The respective reciprocal cross did not affect the phenotypes at a population level, as no significant differences were seen between the phenotypic mean values between cross 1 and cross 2 in either males or females (Table 1). Instead, the reciprocal effects were seen as interactions with specific QTLs and thus provide support for nuclear-mitochondrial interactions to be involved in the genetic regulation of bone phenotypes. Notwithstanding the mounting evidence for mitochondrial interactions involved in complex diseases such as osteoporosis and type-2 diabetes, other factors such as genomic imprinting, the presence of QTLs on sex chromosomes and maternal environment, are theoretical explanations for reciprocal cross specific inheritance and may contribute to the observed effects as previously discussed [23]. Additionally, since all cross-specific QTLs in this study were identified in males, we cannot exclude the possibility that interactions with a Y chromosome linked locus could explain the observed reciprocal cross effect. However, the more than 100 variant positions in both coding and non-coding regions that have been identified in GK mitochondrial DNA compared to F344 [25], [26], support mitochondrial genotype as a possible factor behind the observed reciprocal cross effect in this study. The reciprocal cross interactions could thus reflect functional interaction between nuclear- and mitochondrial encoded proteins. Although the candidate regions are large and contain many genes, it is interesting to note that the QTL regions on chromosome 4 and 6 that demonstrated significant reciprocal cross interaction contain genes encoding mitochondrial proteins. These include mitochondrial ribosomal protein S33 [Mrps33], the NADH dehydrogenase (ubiquinone) 1 beta subcomplex 2 [Ndufb2] and glutathione S-transferase kappa 1 [Gstk1] on chromosome 4 and ATP synthase subunit s [Atp5s] and Acyl-coenzyme A thiosterase 2 [Acot2] on chromosome 6. In addition, the region on chromosome 4 overlaps a previously identified QTL strongly linked to femoral neck structure phenotypes in female (F344×LEW) F2 rats [14].

Several QTLs identified in this study co-localized with our previously reported QTLs linked to tibial bone phenotypes obtained from pQCT and 3D CT [23], [24] and were in many cases influenced by sex- and reciprocal cross (Table 4). The region on chromosome 1 (17–79 cM) linked to multiple phenotypes obtained from all four methods (pQCT, 3D CT, 3PB and DXA) in both males and females showed predominantly higher significance in the reciprocal cross 1 carrying GK mtDNA. Several QTLs linked to cortical bone traits in other combinations of rat strains have been mapped to this region [14], [16], emphasizing the importance of this large chromosome region for variation in bone traits between individuals. The osteoporosis candidate genes transforming growth beta 1 (TGFB1) [36] and estrogen receptor alpha (ESR1) [37] are both localized within this locus. Interestingly, QTLs for fasting glucose also map to this region [38] making it a strong candidate for focused investigation of possible shared or linked polymorphisms regulating diabetes- and osteoporosis-related phenotypes. To clarify shared mechanisms between diabetes and osteoporosis, such analyses could include congenic strains and/or advanced intercross lines. It is recognized, as a possible limitations, that this chromosomal region is large and likely to harbor several sub-loci.

Several established osteoporosis candidate genes are also found within the human regions syntenic to the chromosome 4 QTL linked to multiple rat bone strength phenotypes. These include the calcitonin receptor (7q21.3), collagen 1 alpha 2 (7q21.3) and WNT 2 and WNT 12 from the WNT signalling pathway. The cytokine macrophage migration inhibitory factor (MIF) gene, recently shown to be associated with bone loss in elderly women [39] is also located within this region. The human regions syntenic to the rat chromosome 6 QTL contains around 140 genes. This region is too large to pinpoint candidate genes, but interestingly contains genes which contribute to skeletal development e.g. the transcriptional regulator TWIST1 (7p21) and GDF7 (2p24), a member of the TGFB superfamily. Further delineation of the complex genetic architecture of bone phenotypes for fracture susceptibility would ideally involve a genome screen for epistatic interactions between the identified loci. However, such analysis requires a larger sample size than was available in this study. Congenic strains or advanced intercross lines are possible tools for future fine mapping of the identified QTLs, and would allow for positional cloning of candidate genes and epistatic interaction analyses, respectively.

In summary, we have identified QTLs for biomechanical and two-dimensional DXA phenotypes influencing bone strength with interaction from reciprocal cross in an F2 intercross between GK and F344 rats. The observed interaction between nuclear QTLs and reciprocal cross for numerous bone associated phenotypes supports the potential for mitochondrial effects on bone. By combining data from this and our previous studies, we were able to identify specific but also overlapping chromosomal regions for bone size, structure and strength. These findings illustrate the importance of analysing different determinants contributing to bone strength in order to detect candidate genes or pathways for bone regulation, from the macro- to the micro-structural level. Of particular interest is the identified QTL region on chromosome 1 linked to many bone phenotypes and also reported to affect fasting glucose. This region is thus a strong candidate for the identification of genes contributing to bone regulation and potentially type-2 diabetes.

## Supporting Information

Table S1 **QTL size defined as the region covered by a 1-LOD reduction for any of the bone traits.** ^a^Genome-wide suggestive QTLs (LOD≥2.3) are marked in bold.
(DOC)
