# Supplementary material for: Identification of Candidate Gene Regions in the Rat by Co-Localization of QTLs for Bone Density, Size, Structure and Strength
Source: PLoS One. 2011 Jul 27;6(7):e22462. doi: 10.1371/journal.pone.0022462 (PMC3144887; doi:10.1371/journal.pone.0022462)
Supplement: Table S1 — QTL size defined as the region covered by a 1-LOD reduction for any of the bone traits. aGenome-wide suggestive QTLs (LOD≥2.3) are marked in bold. (DOC) [file pone.0022462.s001.doc]

**Table S1.** Suggestive QTLs for biomechanics and DXA phenotypes in male and female F2 rats

|  |  |  |  |  | **LOD scores** | | |
| --- | --- | --- | --- | --- | --- | --- | --- |
|  | **Chr** | **QTL region** | **Position (cM)** | **Phenotype** | **Male (n=108)** | **Cross 1 (n=66)** | **Cross 2 (n=42)** |
| **Biomechanics** | 1 | D1Wox16-D1Mgh1 | 48-79 | Ult force | **2.3** | **2.8** | 0.9 |
|  | 1 | D1Mgh7-D1Mgh34 | 71-110 | Stiffness | **2.5** | **3.5** | 0.9 |
|  | 2 | D2Mit24-D2Rat161 | 49-66 | Ult force | 1.4 | 0.1 | **2.8** |
|  | 3 | D3Mit10-D3Rat189 | 3-24 | Ult force | **2.9** | **2.5** | 1.4 |
|  | 18 | D18Mit13-D18Mit2 | 0-11 | Work to fail | 1.1 | **3.2** | 0.2 |
|  | 19 | D19Mit15-D19Mit10 | 0-23 | Stiffness | 2.1 | 0.7 | **2.8** |
| **DXA** | 1 | D1Mgh1-D1Wox22 | 78-121 | aBMD | **2.4** | 1.8 | 0.8 |
|  | 5 | D5Mgh21-D5Mgh16 | 92-109 | aBMD | 0.4 | 1.1 | **2.8** |
|  | 10 | D10Mgh5-D10Mit11 | 73-91 | aBMD | **2.4** | 1.5 | 1.1 |
|  | 12 | D12Rat23-D12Rat15 | 29-49 | BMC | **2.7** | **2.8** | 0.6 |
|  | 17 | D17Rat1-D17Rat8 | 14-35 | aBMD | **3.1** | 1.8 | 1.4 |
|  |  |  |  |  | **Female (n=98)** | **Cross 1 (n=48)** | **Cross 2 (n=50)** |
| **Biomechanics** | 2 | D2Mit1-D2Rat4 | 3-23 | Ult force | 1.8 | **3.3** | 1.4 |
|  | 2 | D2Mit1-D2Rat4 | 2-30 | Stiffness | **2.7** | **3.7** | 0.6 |
|  | 4 | D4Mit9-D4Mit24 | 37-49 | Ult force | 0.5 | **2.5** | 0.2 |
|  | 4 | D4Mit28-D4Mgh7 | 59-74 | Work to fail | 0.8 | **2.5** | 0.3 |
|  | 5 | D5Wox7-D5Mgh24 | 29-54 | Work to fail | 2.0 | **3.0** | 0.2 |
|  | 7 | D7Mit23-D7Mit7 | 18-30 | Ult force | 2.1 | 0.4 | **2.3** |
|  | 8 | D8Mit2-D8Mgh4 | 49-63 | Work to fail | **2.9** | 0.9 | 2.0 |
|  | 10 | D10Mgh27-D10Mit14 | 0-8 | Ult force | 0.7 | 0.6 | **2.3** |
|  | 12 | D12Rat57-D12Mit5 | 9-19 | Stiffness | 1.3 | 0.3 | **2.6** |
|  | 14 | D14Mit17-D14Mit10 | 48-64 | Stiffness | **2.8** | **2.6** | 0.8 |
|  | 19 | D19Mit15-D19Mit10 | 0-8 | Work to fail | 1.7 | **2.8** | 0.1 |
|  | 20 | D20Mit4-D20Rat29 | 34-41 | Stiffness | **2.6** | **2.6** | 0.7 |
| **DXA** | 1 | D1Rat4-D1Mgh2 | 8-27 | aBMD | 1.8 | 0.4 | **2.7** |
|  | 1 | D1Rat7-D1Rat20 | 19-47 | Area | **2.5** | 1.3 | **2.3** |
|  | 1 | D1Rat7-D1Rat20 | 18-45 | BMC | **3.2** | 1.5 | **3.7** |
|  | 1 | D1Wox20-D1Wox22 | 126-140 | Area | **2.5** | **3.1** | 1.1 |
|  | 1 | D1Mgh13-D1Mgh15 | 163-169 | BMC | 1.8 | **2.4** | 0.7 |
|  | 9 | D9Rat4-D9Rat1 | 75-85 | aBMD | 1.0 | 1.2 | **2.5** |
|  | 12 | D12Rat57-D12Mit5 | 12-19 | Area | 0.8 | 0.2 | **2.3** |
|  | 16 | D16Rat27-D16Rat14 | 30-56 | Area | **2.7** | **2.4** | 1.3 |
|  | 18 | D18Mit13-D18Mit2 | 4-24 | aBMD | 1.8 | **2.4** | **2.3** |
